# Using a combination of superb microvascular imaging and other auxiliary ultrasound techniques to increase the accuracy of gray-scale ultrasound for breast masses

**DOI:** 10.1186/s12885-024-11981-9

**Published:** 2024-02-16

**Authors:** Mahboubeh Abedi, Leyla Sahebi, Bita Eslami, Azin Saberi, Marzieh Orouji, Sadaf Alipour, Shirin Shahsavarhaghighi

**Affiliations:** 1 Radiology Fellow, Ballarat Base Hospital, Ballarat, VIC Australia; 2 Maternal, Fetal and Neonatal Research Center, Family Health Research Institute, Tehran University of Medical Sciences, Tehran, Iran; 3 Breast Diseases Research Center, Cancer Institute, Tehran University of Medical Sciences, Imam Khomeini Hospital Complex, Keshavarz Blvd., Tehran, Iran; 4 Department of Surgery, Arash Women’s Hospital, Faculty of Medicine,Tehran University of Medical Sciences, Tehran, Iran; 5 Department of Nursing, Arash Women’s Hospital, Tehran University of Medical Sciences, Tehran, Iran; 6 Faculty of Medicine, Tehran University of Medical Sciences, Tehran, Iran

**Keywords:** Breast Imaging, Breast Neoplasm, Doppler Ultrasound, Diagnostic Imaging, Elastography, Vascular flow

## Abstract

**Background:**

Breast ultrasound is highly sensitive, but its specificity is not as high for detecting malignant lesions. Auxiliary modalities like elastography, Color and Power Doppler ultrasound are used as adjuncts to yield both a high sensitivity and specificity. Superb microvascular imaging (SMI) is a newer modality with more accuracy for detecting breast lesions. In this study, our goal was to investigate the role of SMI as an adjunct to ultrasound and find a suitable combination model for the evaluation of breast masses.

**Methods:**

In this cross-sectional study, 132 women with 172 breast masses who underwent ultrasound-guided biopsy were included.. The ultrasound features of the lesion, the strain ratio in strain elastography, the number of vessels for each lesion, their morphology and distribution in Doppler and Power Doppler ultrasound and SMI were recorded for each lesion. A vascular score and a vascular ratio were defined.

**Results:**

In the histologic examination, 31 lesions (18%) were malignant and 141 lesions (82%) were benign. The vascular score was more accurate than the vascular ratio in all three modalities. The predictive ability of strain ratio was higher than Doppler and Power Doppler ultrasound and SMI. Adding SMI alone to ultrasound increased the specificity from 46.10% to 61.2% and the accuracy from 55.80% to 70.11%. In the combination of ultrasound with other modalities, the best was the combination of ultrasound, strain elastography, and SMI; which yielded a specificity and sensitivity of 100% and 74.4%, respectively.

**Conclusion:**

Adding SMI and STE modalities as adjuncts to ultrasound lowers the chance of missing malignant lesions and reduces unnecessary biopsies of breast lesions. A study with a larger sample size using this combination model to evaluate the accuracy with greater precision is recommended.

## Introduction

Breast lesions are usually assessed by mammography, ultrasound (US), or MRI, depending on the characteristics of each patient. Breast US can assist in differentiating benign from malignant lesions based on features like shape, echogenicity, margins, or posterior features. These findings sum up in a Breast Imaging-Reporting and Data System (BIRADS) assessment category (according to the American College of Radiology; ACR) assigned by the radiologist which alludes to the amount of suspiciousness [1].

Some auxiliary modalities have been developed as diagnostic adjuncts to increase the accuracy of grayscale US. Strain elastography (STE) estimates the elasticity of the lesion and adds to the specificity of US [2, 3]. Other assisting technologies detect new vessels in the region. Neovascularization is a key event in the development of malignancy; small branches grow from vessels in and around and advance into the emerging neoplasm [4, 5]. Doppler US (DUS) is widely used for investigating the vascularity of breast lesions US [6]. However, it cannot detect vessels less than 0.1 mm, is angle-dependent, and is not capable to differentiate between low-volume blood flow and tissue movements (cluttering) in the breast tissue; eliminating these features while providing the flow image [7, 8]. Power Doppler US (PUS) is more sensitive than DUS for the detection of low-volume, slow blood flow, and is not angle-dependent; it displays more definite criteria than DUS to differentiate malignant from benign lesions [9, 10].

In these modalities, a higher vessel number is a criterion in favor of malignancy. For the distribution of vessels, peripheral vascularity is less suspicious than central vascularity, and their combination is worse [7]. Four types of morphologies have been defined: dot-like, linear, branching, and penetrating, respectively named types 1 to 4; showing an increasing level of suspiciousness [7].

A more recent technique is superb microvascular imaging (SMI), which filters tissue cluttering, and distinguishes the sluggish blood flow in small vessels [11]. The first work about breast SMI was published in 2015 [12], and then several studies investigated its capabilities reporting that SMI shows the number, distribution and morphology of vessels in detail; even more specifically than contrast-enhanced studies [12, 13].

Two very recent systematic reviews have been carried out on this topic [8, 13], suggesting that SMI might be superior to other supplementary modalities in the differentiation of benign and malignant lesions. Nevertheless, their collective sample size was not large enough, and non-biased studies suitable for the meta-analyses were limited to three countries only (China, Korea, and Turkey). Also, studies about the results of the combination of US, SMI and other auxiliary methods are scarce. Therefore, we conducted this study to investigate the role of SMI as a supplementary measure to US, and also find and present a sensitive and specific novel combination model comprising gray-scale US and these auxiliary modalities for evaluation of breast masses.

## Materials and methods

### Settings, participants, variables and outcomes

This cross-sectional study was approved by the Ethics Committee of Tehran University of Medical Sciences (TUMS), Ethics Code IR.TUMS.IKHC.REC.1399.428. It was held from July 2020 to September 2021 at Arash Women’s Hospital, affiliated with TUMS. The study population consisted of the patients attending the Breast Clinic. All participants signed a written informed consent.

Women aged above 20 with one or more breast masses on US who needed to undergo US-guided biopsy according to the breast surgeon's request were included. Exclusion criteria were pregnancy, previous breast surgery or radiation, and inflammatory signs; because these affect vascularity.

The breast surgeon recorded mass palpability. The breast clinic nurse recorded the personal, demographic, anthropometric, and reproductive features of participants. Then breast grayscale US, DUS, PUS, STE, and SMI were done for all participants by the radiologist. Tumor characteristics including the size, number, architecture and vessel distribution; and STE values were recorded. When a color focus was suspected to be an artifact, its vascular flow was checked with pulsed Doppler to differentiate between real vessels and artifacts. Thereafter, US-guided core needle biopsy was performed by using a 14-Gauge automated gun (Max-Core gun; Bard, Covington, GA, USA) in all cases. The tissue samples were immediately fixed in formalin and sent to the laboratory for histological assessment.

Our main outcomes consisted of the accuracy, sensitivity (SE), specificity (SP), predictive values (PVs) and likelihood ratios (LRs) of SMI vs. DUS, PUS and STE versus histological assessment for differentiating benign from malignant breast masses. SE, SP, and PVs, and LRs of some combination models were our secondary outcomes.

### Measurements and categorization of variables

During the US examinations, patients were placed in the supine or supine semi-oblique position, arms elevated. Breast US and DUS, PUS, STE, and SMI were carried out by one breast-dedicated, board-certified radiologist with ten years of experience in breast US imaging by using an Aplio 500 Platinum ultrasound unit (Toshiba Medical System, Tokyo, Japan) with high‑frequency (14 MHz) linear array transducers. The settings for DUS and PUS included frame rate 7–11, dynamic range 65, velocity scale 3.1–4.4 cm/s, wall filter 3–5. The technical settings of SMI were frame rate 26–50, dynamic range 65, velocity scale 1–1.9 cm/s, wall filter 0; minimal transducer pressure was used to preserve the small vessels flow. During STE, the strain ratio (SR) was obtained from the region of interest (ROI) in the mass and the tissues surrounding it. The radiologist performed the methodology seven times for each patient; this took from 5–15 min.

A BIRADS score from 1 to 5 was assigned by the radiologist on grayscale US examination according to the lesion size, depth, shape, margin, echogenicity, and posterior acoustic elements findings, based on the ACR BIRADS classification [1].

**Fig. 1:**
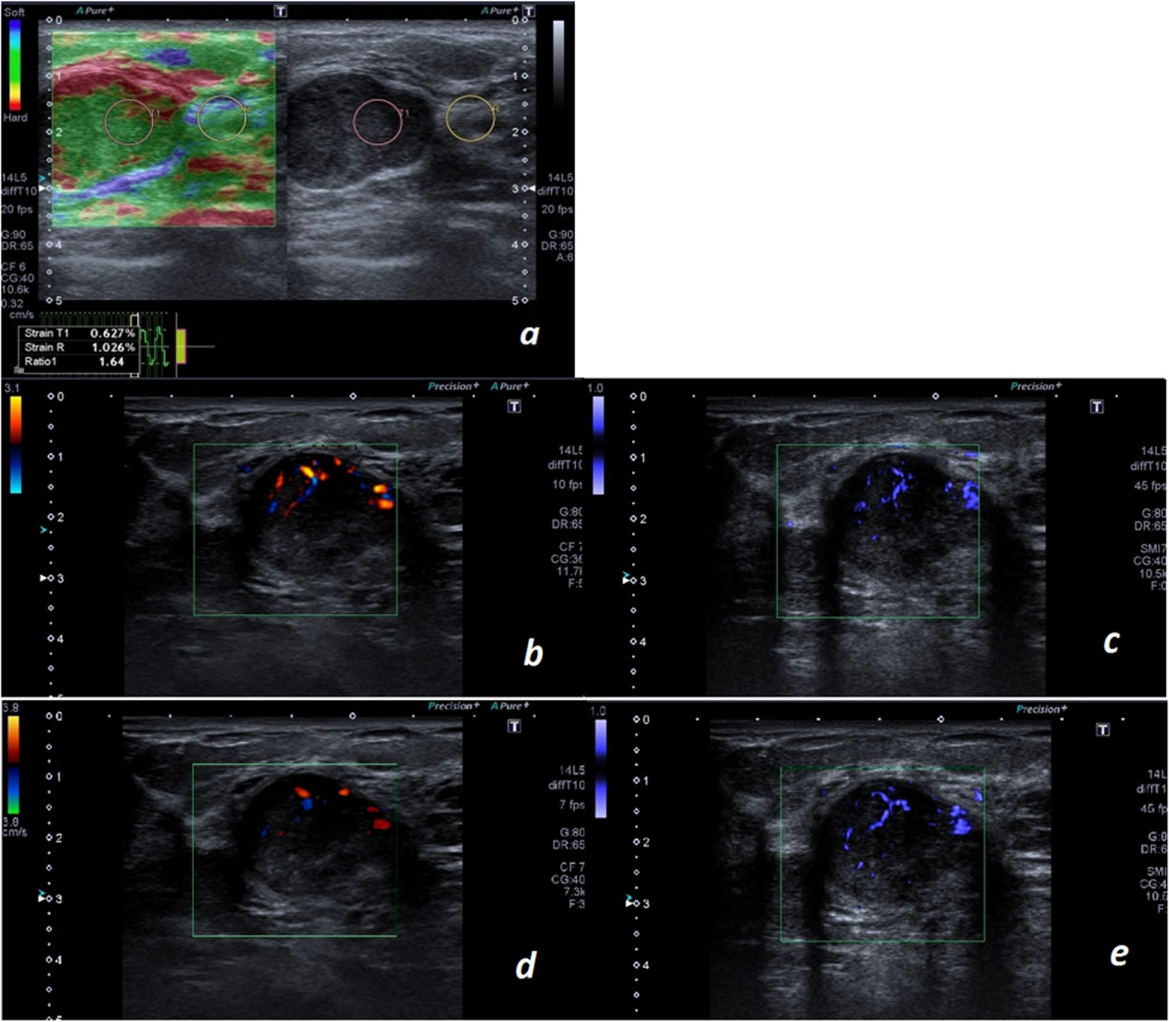
Pathologically proved fibroadenoma. **a** STE shows a low strain ratio. DUS (**b** and **d**) and SMI (**c** and **e**) at two different sections of the mass. DUS shows a few dot-like and linear vessels within the mass. SMI shows a higher number of vessels within the lesion and provides more detailed information about the vessels 'morphology including detection of one branching pattern vessel. Also SMI is superior in detecting vessels in the deeper portions of the mass

**Fig. 2:**
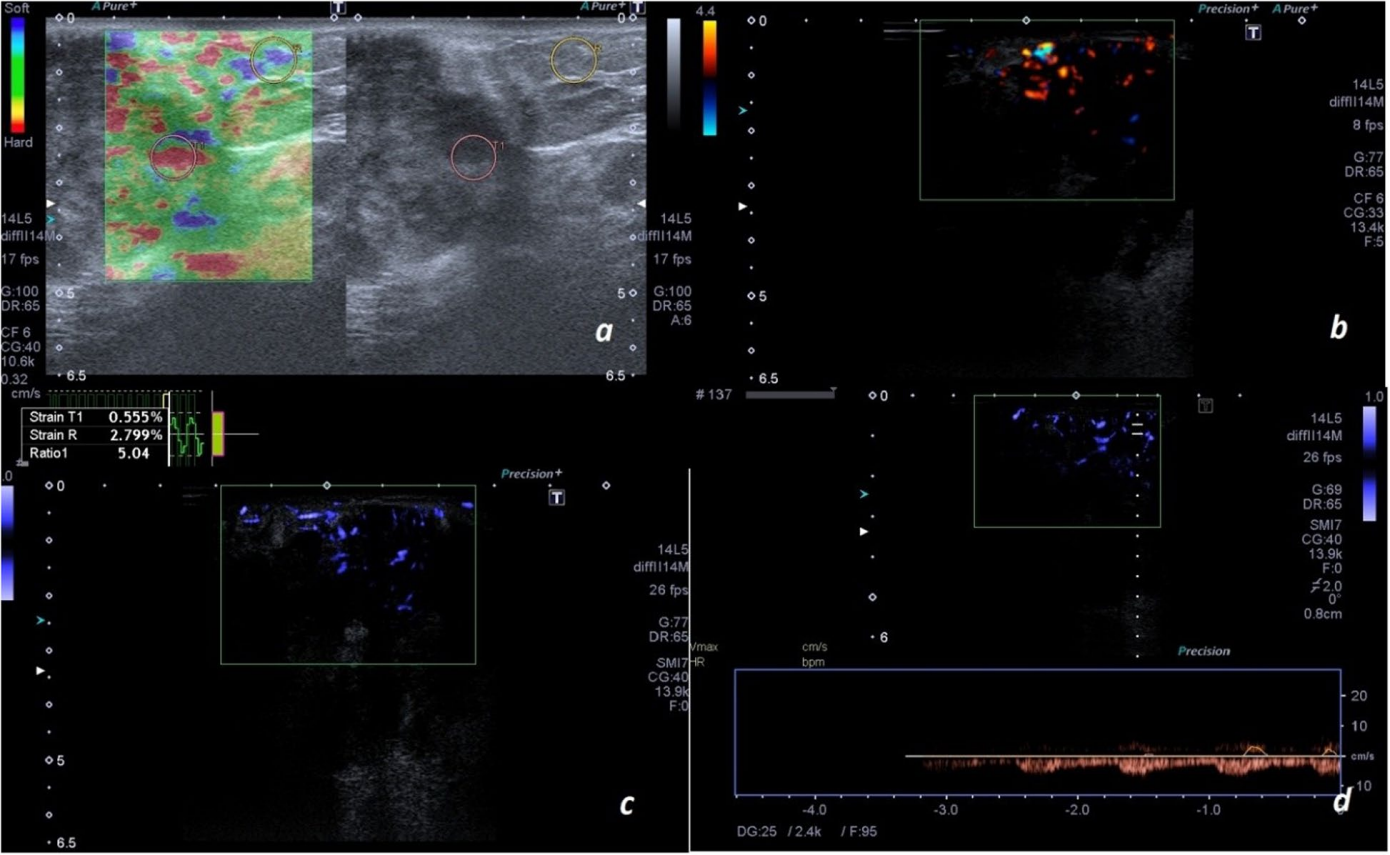
Pathologically proven invasive ductal carcinoma. **a** STE shows a high strain ratio. Both DUS (**b**) and SMI (**c**) show multiple dot-like and linear vessels within the mass. However, SMI shows a higher number of vessels within the lesion in comparison to DUS. Especially, SMI is superior in showing the vessels of the deeper portion of the mass. **d** There was arterialized flow on the spectral Doppler examination

During DUS, PUS and SMI, the vessels’ number, morphology and distribution were recorded by the radiologist (Figs. 1 and 2). Vessels’ morphology was categorized into four groups including dot-like, linear, branching, and penetrating; and vascular distribution was classified as peripheral (all vessels located at the margin of the lesion), central (all vessels located within the mass without extension to periphery), or both [7]. For STE, SR was categorized according to the cut-point found in the present study.

We did not have the vascular index (VI) of the lesions, which is computed as the ratio of color to all pixels within a lesion [14]. However, since the number of vessels relative to the size of the lesion is rationally more important than the absolute number, we defined the “vascular ratio” (VR) as a new variable by dividing the number of detected vessels by the largest dimension of the lesion on US. We estimated the VR in DUS, PUS, and SMI.

**Fig. 3:**
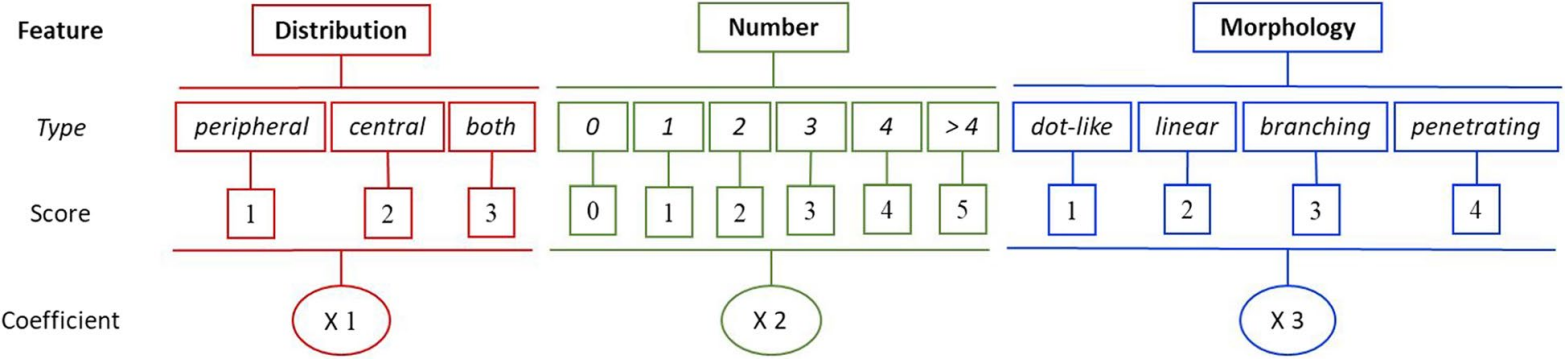
Scoring system of vascular findings in color Doppler ultrasound, power ultrasound, and SMI

We also defined a vascular score (VS) for DUS, PUS and SMI each, to quantify the results of vascular findings as a single indicative value. For this, we used the scoring defined by Park et al. [7], who defined a score by considering vascular findings of SMI. They rated the number of vessels from 0 to 5 according to the same number of vessels, and 6 for those with 6 or more vessels. They gave a score from 1 to 4 for the 4 types of morphology, respectively. They also assigned score 1 to peripheral distribution, 2 to central, and 3 to both. The scores were finally summed, and a score from 0 to 13 was allocated to the SMI of each lesion. We used this system, but assigned coefficients to give more weight to the more important variables according to our radiologist’s experience; and considered our results for defining the upper range in the number of vessels. Figure 3 shows our scoring system.

In order to find the best combination of modalities, as the SE of US alone was high enough, we aimed to explore the combination of US with modalities that had higher SP; in order to obtain a combination with a higher SP then US alone, while preserving the SE of US. We thus envisaged several combinations of the modalities with higher SPs and high SEs and accuracies, and calculated the diagnostic performances of the combinations, to find the one that could increase the diagnostic performance of gray-scale US alone in differentiating malignant from benign breast lesions.

For histology, malignant lesions included all types of breast carcinoma consistent with the WHO classification of tumors [15]. Premalignant lesions including atypia, papillomas, and benign lesions were all categorized as benign.

### Bias

To maintain consistency, all vascularity measurements in various flow modes were performed by one radiologist with one device. The radiologist was blind to the histopathology results. To address a selection bias, we included all the lesions that needed biopsy during the study period, although this caused heterogeneity in the number of benign and malignant lesions.

### Study size and power

We calculated the sample size according to a 5-year prevalence of 37% for BC in Iran [16], a sensitivity of 81% and a specificity of 71% for SMI in the evaluation of breast masses [13]. Considering a power of 90% and α = 0.05, a sample size of 160 masses was obtained by using the Sample Size Calculator available at https://wnarifin.github.io/ssc/sssnsp.html.

### Statistical methods

Statistical analysis was performed by using SPSS (IBM Corp. 2016. IBM SPSS Statistics for Windows, version 24.0. Armonk, NY: IBM Corp). *P*-Values < 0.05 were considered significant. Continuous variables are presented as mean ± standard deviation, and categorical variables are presented as numbers (and percentages).

The VS of DUS, PUS, and SMI were calculated and used as the quantitative value in further analyses; while SR was considered for STE. We used receiver operator characteristics (ROC) analysis to estimate the ability of DUS, PUS, SMI, and Elastography, alone and combined together to predict the benign vs. malignant status of the breast lesion by the area under the curve (AUC). The optimal cut-off value was defined as maximizing the Youden index (sensitivity + specificity – 1) index. Positive predictive value (PPV), negative predictive value (NPV), positive likelihood ratio (PLR), negative likelihood ratio (NLR), and accuracy of detection techniques were calculated by considering the histopathologic diagnosis as the reference standard. For the combination of the modalities, considering the sensitivity and specificity of US, we aimed to combine it with a modality with a high SP. We chose among the modalities that had the highest specificities when used alone, in addition to a high accuracy and sensitivity. Then, the models that could yield accurate and specific results while saving the sensitivity were sought among these options in combination with US. The combinations of modalities consisted of considering a positive or negative result when all modalities were positive, or all were negative, respectively.

## Results

### Participants and descriptive data

**Fig. 4:**
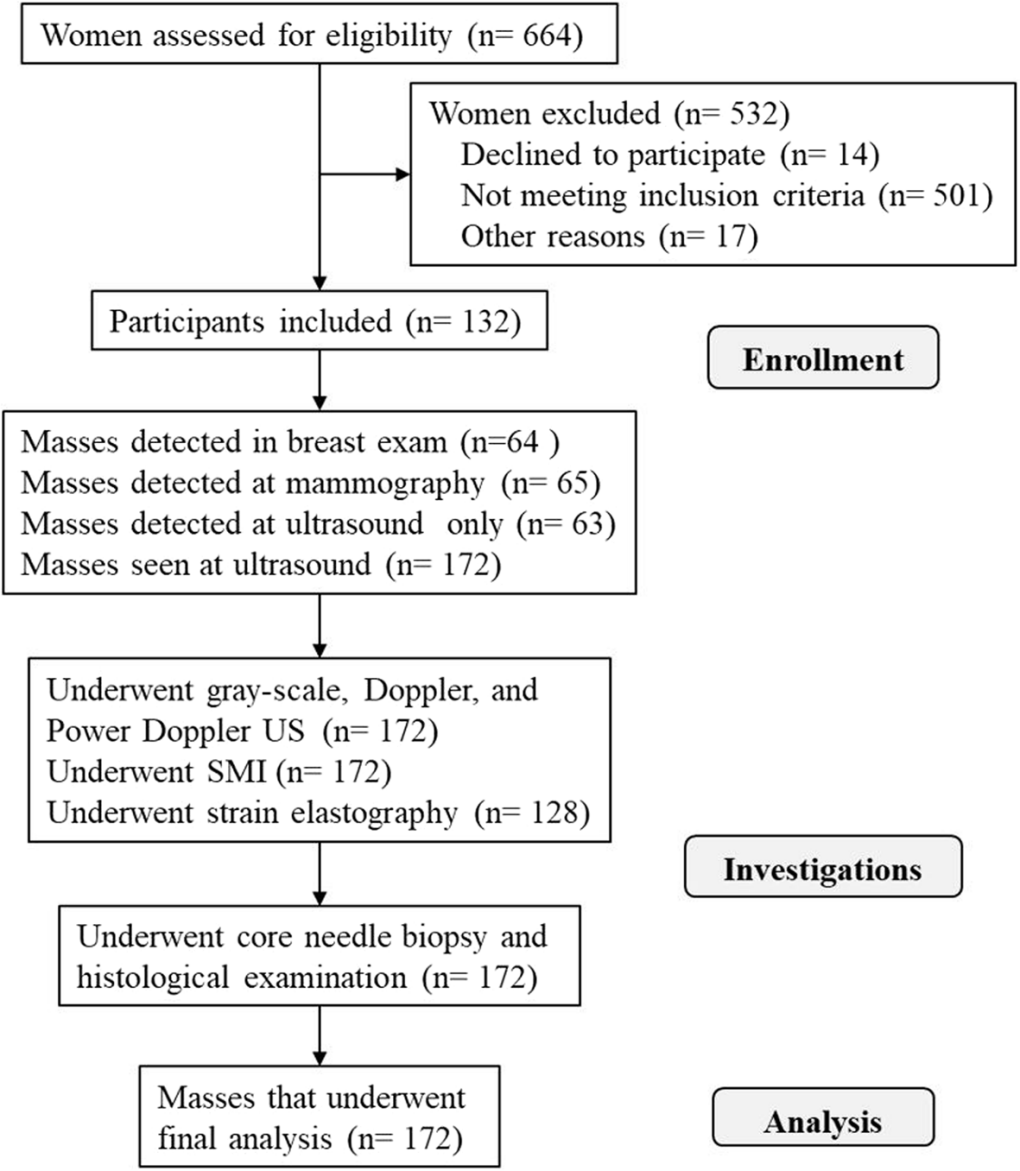
Flow diagram of all participants

Overall, 132 patients with 172 breast masses were included. The flow diagram of participants is shown in Fig. 4, and their characteristics are demonstrated in Table 1. In the histologic examination, 31 lesions (18%) were malignant and 141 lesions (82%) were benign. The average size of the malignant masses was 21.67 ± 11.24 mm and that of benign masses was 16.5 ± 8.8 mm (*p* = 0.008), thus the difference in mass size between the two groups was significant.

**Table 1:** Characteristics of all the participants based on tumor histology

Variables		Mean (SD) or Number (%)						*P*-value
		Total	N	Benign mass	N	Malignant mass	N	
Age		41.93 (11.43)	132	40.58 (11.07)	111	49.67 (10.72)	21	0.001
BMI		26.53 (4.38)	121	25.99 (4.42)	101	27.92 (4.41)	20	0.076
Menopause	No	100		90 (90.0)		10 (10.0)		< 0.001
	Yes	34		21 (61.8)		13 (38.2)		
Family history	No	100		85 (85.0)		15 (15.0)		0.821
	Yes	30		26 (86.7)		4 (13.3)		
Mass palpability	No	108		98 (90.7)		10 (9.3)		< 0.001
	Yes	64		43 (67.2)		21 (32.8)		

*N* Number of patients in that subgroup, *SD* Standard deviation

The distribution of histologies among US-BIRADS and the SR of STE are demonstrated in Table 2. The vascular features detected on DUS, PUS, and SMI are demonstrated in Table 3.

**Table 2:** BIRADS categories of breast ultrasound and strain ratios of strain elastography according to histology results

Histology		Benign		Malignant		Sum	
Variables		Number	Percent	Number	Percent	Number	Percent
US-BIRADS (*N* = 172)	B1	0	0.0	0	0.0	0	0.0
	B2	1	0.7	0	0.0	1	0.6
	B3	64	45.4	0	0.0	64	37.0
	B4a	65	46.1	1	3.2	66	38.2
	B4b	9	6.4	5	16.1	14	8.1
	B4c	2	1.4	13	41.9	15	8.7
	B5	0	0.0	12	38.7	12	6.9
		Mean (SD)	Median (Range)	Mean (SD)	Median (Range)	Mean (SD)	Median (Range)
STE (*N* = 128)	Strain ratio (kPa)	2.69 (1.19)	2.44 (1.02–7.13)	4.74 (2.41)	4.28 (1.91–10.16)	2.94 (1.53)	2.58 (1.02–10.16)

*STE* Strain elastography, *US* Ultrasound

**Table 3:** Vascular findings in all breast lesions on color Doppler ultrasound, power ultrasound, and SMI according to histology results

Variable		Histology	Color Doppler Ultrasound		Power Ultrasound		SMI	
			Mean (SD)	Median (Range)	Mean (SD)	Median (Range)	Mean (SD)	Median (Range)
Vessel number		B	3.60 (5.47)	1 (0–35)	5.66 (8.19)	2 (0–45)	5.86 (8.76)	1 (0–50)
		M	6.65 (5.06)	6 (0–15)	10.97 (9.07)	8 (0–27)	10.97 (9.07)	8 (0–27)
Variable			Number (%)		Number (%)		Number (%)	
Vessel morphology	T1	B	41 (59.4)		46 (57.5)		46 (62.2)	
		M	14 (58.3)		14 (56.0)		16 (59.3)	
	T2	B	62 (89.9)		74 (92.5)		68 (90.7)	
		M	22 (91.7)		22 (88.9)		24 (88.9)	
	T3	B	30 (43.5)		36 (45.0)		33 (44.0)	
		M	8 (33.3)		10 (40.0)		11 (40.7)	
	T4	B	21 (30.9)		30 (37.5)		28 (38.4)	
		M	11 (45.8)		14 (56.0)		13 (48.1)	
Vessel distribution	C	B	0 (0.0)		0 (0.0)		0 (0.0)	
		M	0 (0.0)		0 (0.0)		0 (0.0)	
	P	B	12 (21.1)		16 (25)		16 (25.8)	
		M	2 (8.7)		3 (12.5)		5 (20.8)	
	C&P	B	45 (78.9)		48 (75.0)		46 (74.2)	
		M	21 (91.3)		21 (87.5)		19 (79.2)	

The Number of cases without vessels were 69, 61 and 65 on Color Doppler Ultrasound, Power Ultrasound, and SMI, respectively

*B* Benign, *C* Central, *M* Malignant, *N* Total number, *NA* Not applicable, *P* Peripheral, *SD* standard deviation, *T* Type

### Outcome data and main results

**Fig. 5:**
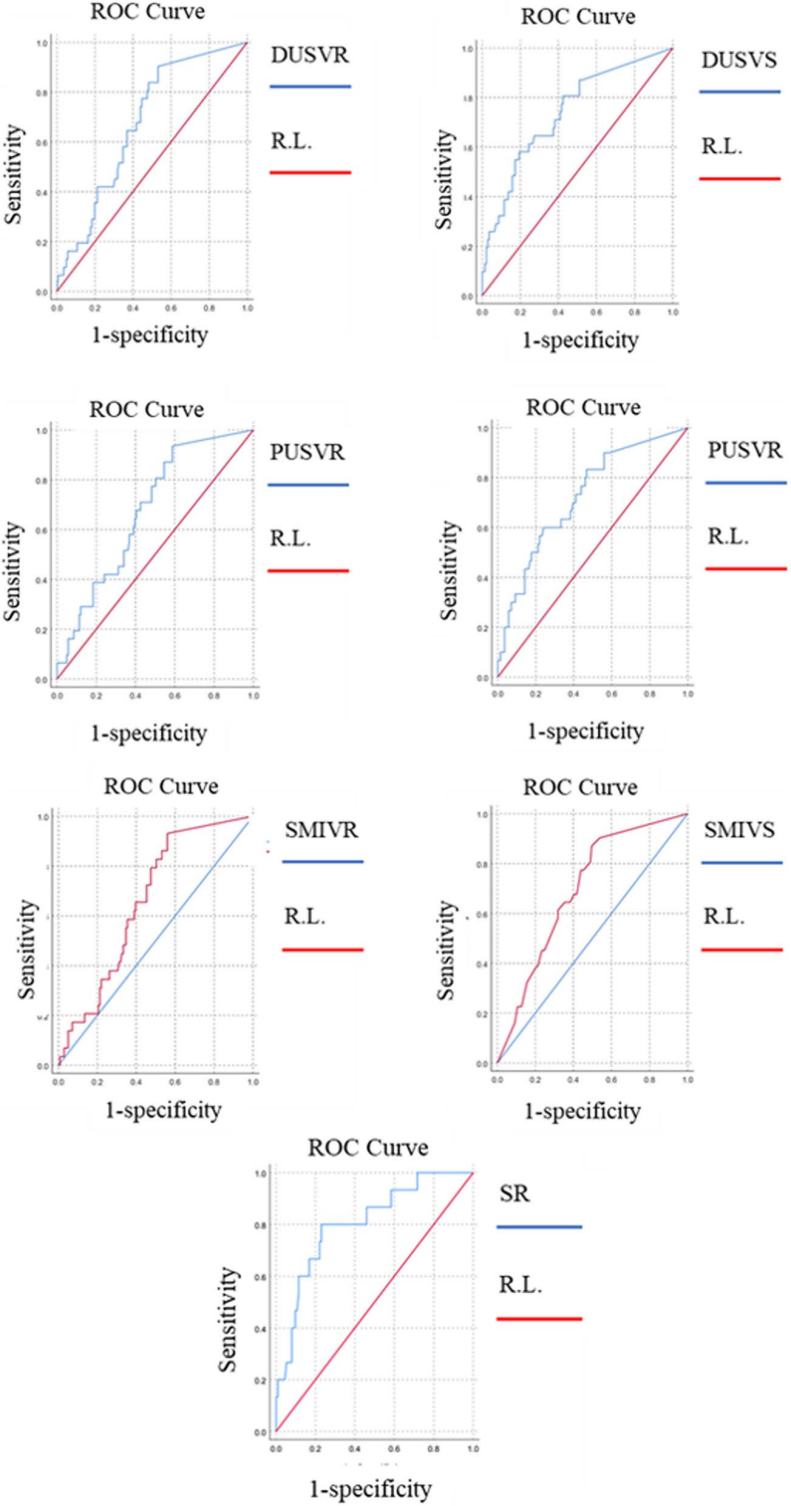
ROC curve of the diagnostic performance of strain elastography and the vascular scores and ratios of superb microvascular imaging, Doppler and Power ultrasound for breast masses (DUS = Doppler and Power ultrasound, PUS = power ultrasound, R.L. = Reference Line, SMI = superb microvascular imaging, SR = strain ratio, VR = vascular ratio, VS = vascular score)

The predictive ability of SR (AUC = 0.805) was higher than that of DUS VS and DUS VR (AUC s = 0.739 and 0.676), PUS VS and PUS VR (AUC s = 0.726 and 0.675), and SMI-VS and SMI VR(AUC s = 0.696 and 0.66) respectively. The AUC of 0.805 for SR indicates that the STE has a strong predictive power in the evaluation of breast masses. Also, VS was more accurate than VR in all three modalities (Fig. 5).

The cut-off point, SE, SP, PPV, NPV, PLR, NLR of VR and VS of DUS, PUS and SMI and SR of STE, and these values for our breast US as well as the combination models are presented in Table 4. Due to the 100% SE but low SP of US BIRADS, we combined it with modalities with the highest specificities in addition to high accuracies and sensitivities (Table 4). The addition of SMI to US increased the SP from 46.1% to 61.2%, with no change in the SE, and the addition of STR to US augmented the SE to 79.3%; but decreased the SE from 100 to 90%. Among all the dual and triple combinations, US + SMI + SR showed the highest values (Table 4) by maintaining a 100% sensitivity and increasing the specificity from 46.1% to 74.4%.

**Table 4:** Sensitivities, specificities, predictive values, and likelihood ratios of breast ultrasound BIRADS, vascular ratios and scores of SMI, color Doppler ultrasound and power ultrasound; and combination models for all breast lesions

	AUC	SE (AUC)	Cutoff points	SE (%)	SP (%)	PPV (%)	NPV (%)	PLR (%)	NLR (%)	Accuracy (%)
US BIRADS	---	---	---	100	46.10	29.00	100	1.86	0.00	55.80
SMI VS	0.696	0.047	28.5	64.50	65.50	28.60	89.20	1.82	0.55	64.53
SMI VR	0.660	0.047	0.2303	67.70	60.28	27.27	89.47	1.71	0.54	61.63
DUS VS	0.739	0.050	39.3	70.97	61.87	29.33	90.53	1.86	0.47	63.53
DUS VR	0.676	0.047	0.2074	64.52	63.12	27.78	89.02	1.75	0.56	63.37
PUS VS	0.726	0.050	52.0	70.00	60.28	27.27	90.43	1.76	0.50	61.99
PUS VR	0.675	0.048	0.269	64.52	60.28	26.32	88.54	1.62	0.59	61.05
Strain ratio	0.805	0.059	3.09	90.00	62.14	33.75	96.67	2.38	0.16	67.06
US + SMI	*-*	*-*	*-*	100	61.2	43.5	100	2.58	0.00	70.11
US + DUS	*-*	*-*	*-*	100	63.9	46.3	100	2.77	0.00	72.50
Strain ratio + US	*-*	*-*	*-*	90.0	79.3	48.2	97.4	4.34	0.13	81.18
Strain ratio + SMI	*-*	*-*	*-*	94.4	74.4	45.9	98.3	3.68	0.07	78.12
US + DUS + SMI	*-*	*-*	*-*	46.3	100	100	63.9	∞	0.54	72.50
US + STE + DUS	*-*	*-*	*-*	65.8	100	100	59.4	∞	0.34	77.19
US + STE + SMI	*-*	*-*	*-*	100	74.4	63.0	100	3.90	0.00	82.14

Those with no vessels got a zero value. In the combination models: for DUS and SMI, the vascular score; for STE, the strain ratio; and for US, BIRADS is considered. The combinations of modalities consist of considering a positive or negative result when all modalities were positive, or all were negative, respectively

*AUC* Area under the curve, *DUS* Color Doppler ultrasound, *NLR* Negative likelihood ratio, *NPV* Negative predictive value, *PLR* Positive likelihood ratio, *PPV* Positive predictive value, *PUS* Power ultrasound, *SE* Sensitivity, *SMI* Superb microvascular imaging, *SP* Specificity, *STE* Strain elastography, *VR* Vascular ratio, *VS* Vascular score

## Discussion

We carried out a study to examine SMI accuracy and find a combination to better predict the malignant nature of breast masses during US. Our results showed no privilege for SMI alone, but a good predictivity when combining SMI, STE, and US BIRADS.

Breast US has a high SE (100% in our study) but a low SP, which adjunct modalities try to increase [7]. Diao et al. showed a higher accuracy for SMI + US than PUS + US, similar to us [17]. Lee et al. [18] found that the combination of SMI, SR and US upgraded the SP from 46 to 79% while lessening the SE from 98 to 91%. The trend of their findings is similar to ours, but they used VI for SMI; and the sensitivities and specificities were higher in our study. Uysal et al. [19] showed that SR was more accurate than SMI VI. This corresponds with our findings, but we further explored the issue by providing combinations of modalities.

There is no fixed cut-point for the suspicious number of vessels, and there are no quantitative ratings for vascular morphology and distribution; thus no threshold for the vascular findings has been standardized. Park et al. [7] defined the above score to quantify SMI findings, and found higher scores for malignant lesions. Also, they showed that US + SMI was more accurate than US + DUS or US + PUS. However, their score gave equal scores to all findings, whereas our radiologists experience showed that the vascular features do not have equal impacts; and that vessel distribution, number, and morphology have ascending values in favor of malignancy. Therefore, we assigned a coefficient from 1 to 3 to each of these to provide a pertinent weight to that group of findings. We scored morphologic and distributional features as Park et al. [7], and the number of vessels according to our calculated cut-off point of 4 (Fig. 3). Then, we considered findings of DUS, PUS, and SMI as quantitative items and defined the cut-off points. As the accuracy of VS was higher than VR, we considered various combinations of the VRs and SR with US-BIRADS to find the highest accuracy. Considered separately, STE alone had the highest accuracy among the four assisting options (Table 4). When added to US BIRADS, the SP increased to around 79%, but the SE decreased to 90%. The best model was when considering US + STE + SMI. The SE was still 100%, and the SP rose to nearly 75% (Table 4). Thus the combination of the US BIRADS, SR, and SMI lead to a high diagnostic capacity for breast lumps.

Other scoring systems also have quantified vascular findings. Kim et al. [20] assessed 62 lesions (12 malignant) by rating the number of vessels from 0 to 4 (0, 1–2, 3–4, and 5–7 vessels), and the distribution and architecture like Park et al. [7]. Despite the lower sample size, the proportion of malignant to benign masses was close to ours. They found that US + SMI increased the diagnostic accuracy from 66 to 90%, while these were 56% and 70% in our study, respectively. However, we developed these findings by the scorings and the triple combinations, and obtained higher diagnostic yields. Two studies were performed by Zhu et al. [21, 22]. First [21], they showed a SE and SP of 85% for the combination of SMI VI and Virtual Touch Quantification (VTQ) of STE. In the second study [22], they reported an SE and SP of 87% and 85%, respectively, for US + SMI. There, they scored “vascular quantity” as 0–3, morphology as 1–7, and distribution as 1–3. The results of SMI + US in our study were better regarding the SE (100%), but the SP was lower (70.11%). Although the high specificity obtained in their study is very valuable, the lower specificity questions the superiority of the scoring system; and the final combination of US + SMI + STE as proposed in our study presents an obvious advantage. Liang et al. [23] assessed the diagnostic performance of US + SR + SMI. For SMI, they considered the vascular morphology only, and upgraded or downgraded the US-BIRADS according to it. This changed the SP from 44 to 81%, and the SE from 99 to 96%. The main deficit is that it only considered the morphology. In our study, the defined VS was based on definite precise data, was practical and easily calculated, and showed appropriate for the defined goal.

Zhou et al. [24] performed a meta-analysis to evaluate which combination of adjunct modalities was more accurate. The heterogeneity of studies was a serious limitation, and they could not directly envisage a combination of 3 modalities. Also, their approach to BIRADS was clinically impractical because they considered BIRADS 4a, 4b, and 4c as separate entities. Nonetheless, their conclusion was that BIRADS 4b + STE, + SMI was probably more suitable. Although approached differently, this result is in line with ours.

Despite the different settings, the studies that considered several combinations of US and auxiliary modes, including that of Liang et al. [23], Zhou et al. [24], and ours; found the highest diagnostic performance in US + STE + SMI.

Thus, regarding the low SP of US, and the need to auxiliary measures, the combination of US with SMI-VS and STE would yield very accurate results.

Our study had some limitations; the low number of malignant lesions and the lack of VI measurement. Also, the US was done by one radiologist, and the inter-observer difference was not assessed; thus the reproducibility of the study was not explored. In addition, the VR could have been calculated by using the mean or the sum of the dimensions of a mass,instead of the greatest dimension; this could have been a better solution in the case of asymmetric changes.

To conclude, the combination of US, SMI, and STE promotes the diagnostic yield in the differentiation of malignant and non-malignant lesions; increasing the specificity from 46.1% to 74.4%, while maintaining the 100% sensitivity. We propose a study that involves a larger sample size, considers inter-observer variability, and uses VS and the combination model to evaluate the accuracy with greater precision. Two other interesting subjects for future studies would be to compare VS with VI; and the outcomes of using this combination of modalities with the traditional method.

## Data Availability

The data that support the findings of this article are not publicly available due to ethical concerns. They can be requested from the author at sadafalipour@yahoo.com.

## References

[1] Arian A, Dinas K, Pratilas GC, Alipour S. The breast imaging-reporting and data system (BI-RADS) made Easy. Iran J Radiol. 2022;19(1):e121155.

[2] Liu B, Zheng Y, Huang G, Lin M, Shan Q, Lu Y (2016). Breast lesions: quantitative diagnosis using ultrasound shear wave elastography—a systematic review and meta-analysis. Ultrasound Med Biol.

[3] Athamnah SI, Oglat AA, Fohely F (2021). Diagnostice breast elastography estimation from doppler imaging using central difference (CD) and least-squares (LS) algorithms. Biomed Signal Process Control.

[4] Carmeliet P, Jain RK (2000). Angiogenesis in cancer and other diseases. Nature.

[5] Banerjee S, Dowsett M, Ashworth A, Martin L-A (2007). Mechanisms of disease: angiogenesis and the management of breast cancer. Nat Clin Pract Oncol.

[6] Ackerman S (2010). Color Doppler sonography: characterizing breast lesions. Imaging Med.

[7] Park A, Seo B, Woo O, Jung K, Cho K, Park E (2018). The utility of ultrasound superb microvascular imaging for evaluation of breast tumour vascularity: comparison with colour and power doppler imaging regarding diagnostic performance. Clin Radiol.

[8] Feng J, Lu J, Jin C, Chen Y, Chen S, Guo G (2022). Diagnostic value of superb microvascular imaging in differentiating benign and malignant breast tumors: a systematic review and meta-analysis. Diagnostics.

[9] Milz P, Lienemann A, Kessler M, Reiser M (2001). Evaluation of breast lesions by power doppler sonography. Eur Radiol.

[10] Lee SW, Choi HY, Baek SY, Lim SM (2002). Role of color and power doppler imaging in differentiating between malignant and benign solid breast masses. J Clin Ultrasound.

[11] Zhan J, Diao X-H, Jin J-M, Chen L, Chen Y (2016). Superb microvascular imaging—a new vascular detecting ultrasonographic technique for avascular breast masses: a preliminary study. Eur J Radiol.

[12] Ma Y, Li G, Li J, Ren WD (2015). The diagnostic value of superb microvascular imaging (SMI) in detecting blood flow signals of breast lesions: a preliminary study comparing SMI to color doppler flow imaging. Medicine.

[13] Zhong L, Wang C (2020). Diagnostic accuracy of ultrasound superb microvascular imaging for breast tumor: a meta-analysis. Med Ultrason.

[14] Chae EY, Yoon GY, Cha JH, Shin HJ, Choi WJ, Kim HH (2021). Added value of the vascular index on superb microvascular imaging for the evaluation of breast masses: comparison with grayscale ultrasound. J Ultras Med.

[15] Tan PH, Ellis I, Allison K, Brogi E, Fox SB, Lakhani S, Lazar AJ, Morris EA, Sahin A, Salgado R, Sapino A. The 2019 WHO classification of tumours of the breast. Histopathology. 2020;77(2):181–5. 10.1111/his.14091 32056259

[16] Nafissi N (2018). Epidemiology and histopathology of breast cancer in Iran versus other Middle Eastern countries. Middle East J Cancer.

[17] Diao X, Zhan J, Chen L, Chen Y, Cao H (2020). Role of superb microvascular imaging in differentiating between malignant and benign solid breast masses. Clin Breast Cancer.

[18] Lee EJ, Chang Y-W (2020). Combination of quantitative parameters of shear wave elastography and superb microvascular imaging to evaluate breast masses. Korean J Radiol.

[19] Uysal E, Öztürk M, Kilinçer A, Koplay M (2021). Comparison of the effectiveness of shear wave elastography and superb microvascular imaging in the evaluation of breast masses. Ultrasound Q.

[20] Kim ES, Seo BK, Park EK, Woo OH, Jung K, Cho KR (2018). Significance of microvascular evaluation of ductal lesions on breast ultrasonography: influence on diagnostic performance. Clin Imag.

[21] Zhu Y-C, Zhang Y, Deng S-H, Jiang Q (2018). Diagnostic performance of superb microvascular imaging (SMI) combined with shear-wave elastography in evaluating breast lesions. Med Sci Monit.

[22] Zhu YC, Zu DM, Zhang Y, Shan J, Shi XR, Deng SH (2019). A comparative study on superb microvascular imaging and conventional ultrasonography in differentiating BI–RADS 4 breast lesions. Oncol Lett.

[23] Liang M, Ou B, Wu J, Xiao X, Ruan J, Tian J (2020). Combined use of strain elastography and superb microvascular imaging with grayscale ultrasound according to the BI-RADS classification for differentiating benign from malignant solid breast masses. Clin Hemorheol Micro.

[24] Zhou Y, Wu J (2022). Which combination of different ultrasonography modalities is more appropriate to diagnose breast cancer? A network meta-analysis. Medicine.

